# A novel murine in vivo model for acute hereditary angioedema attacks

**DOI:** 10.1038/s41598-021-95125-0

**Published:** 2021-08-05

**Authors:** Sujata Bupp, Matthew Whittaker, Mari Lehtimaki, JuMe Park, Jessica Dement-Brown, Zhao-Hua Zhou, Steven Kozlowski

**Affiliations:** 1 Office of Biotechnology Products, Office of Pharmaceutical Quality, Center for Drug Evaluation and Research, US Food and Drug Administration, 10903 New Hampshire Avenue, Silver Spring, MD 20993 USA; 2 Office of New Drugs, Center for Drug Evaluation and Research, US Food and Drug Administration, Silver Spring, MD 20993 USA

**Keywords:** Experimental models of disease, Complement cascade

## Abstract

Hereditary Angioedema (HAE) is a rare genetic disease generally caused by deficiency or mutations in the C1-inhibitor gene, SERPING1, a member of the Serpin family. HAE results in acute attacks of edema, vasodilation, GI pain and hypotension. C1INH is a key inhibitor of enzymes controlling complement activation, fibrinolysis and the contact system. In HAE patients, contact system activation leads to uncontrolled production of bradykinin, the vasodilator responsible for the characteristic symptoms of HAE. In this study, we present the first physiological in vivo model to mimic acute HAE attacks. We evaluate hypotension, one of the many hallmark symptoms of acute HAE attacks using Serping1 deficient mice (serping1−/−) and implanted telemetry. Attacks were induced by IV injection of a silica nanoparticle (SiNP) suspension. Blood pressure was measured in real time, in conscious and untethered mice using implanted telemetry**.** SiNP injection induced a rapid, reversible decrease in blood pressure, in the presence of angiotensin converting enzyme (ACE) inhibition. We also demonstrate that an HAE therapeutic, ecallantide, can prevent HAE attacks in this model. The in vivo murine model described here can facilitate the understanding of acute HAE attacks, support drug development and ultimately contribute to improved patient care.

## Introduction

The intricate coordination between coagulation and inflammation is the basis for a diverse set of disorders in humans. Hereditary angioedema (HAE[OMIM:106100]) was first described in the early 1800s as a familial form of angioedema, now known to have an incidence of 1:10,000–1:150,000 in the general population. Reports of HAE reflect that for more common forms, prevalence of disease is not affected by ethnicity or sex^1–3^. Patients with HAE typically have intermittent episodes of angioedema of the extremities, gastrointestinal tract, face, larynx or external genitalia, and hypotension^2,4–9^, due to vasodilation and increased vascular permeability. For the vast majority, the physiologic basis of HAE is low-functioning C1-inhibitor protein. A member of the SERPIN (**ser**ine **p**rotease **in**hibitor) superfamily, C1-inhibitor plays a crucial role in regulating contact system activation^10–24^. Unlike histamine-mediated acute allergic reactions, which are usually resolved within 24 h, HAE attacks are histamine-independent and can last for over 72 h^25,26^. HAE can be of multiple types including; type I (85% of cases) and type II HAE (14% of cases), which are generally associated with mutations in the C1INH gene, while HAE with normal C1INH (~ 1% of cases) is idiopathic and not associated with deficiency in levels or function of C1INH, however may be associated with mutations in the FXII gene^27–30^.

Serping1 deficient mice (serping−/−) have been used as a model to better understand the pathophysiology of HAE^31–33^. The readout in these studies was vascular permeability assessed with Evans Blue dye. The role of bradykinin in this model was verified by the dependence on expression of a bradykinin receptor. This model has been used to assess potential therapies; however, increased vascular permeability is a chronic change and the model does not reflect the acute attacks seen in HAE. An HAE animal model that better reflects acute systemic attacks like hypotension, would be useful in assessing both HAE prophylaxis and treatment.

To develop such a model, we considered stimuli of the contact system.

The contact system involves interaction between coagulation and the kallikrein-kinin cascades. In the physiological setting, activation of factor XII and the subsequent conversion of prekallikrein (PK) to kallikrein, stimulates cleavage of high molecular weight kininogen (HK), leading to generation of bradykinin (BK)^34–41^. BK has a short half-life (s) and is catabolized rapidly by carboxypeptidases including angiotensin-converting enzyme (ACE). As ACE plays a pivotal role in degrading bradykinin, ACE inhibitors (ACEi) have been implicated in angioedema, mainly for causing uncontrollable bradykinin generation^42^. While natural biological agents, like RNA^43^, misfolded proteins^44^, collagen^45^ and platelet polyphosphate^46,47^ can cause autoactivation of FXII^48,49^, a diverse array of biomaterial surfaces like glass^50^, dextran sulphate^51^ and silica nanoparticles (SiNPs)^52^ have also been shown to activate FXII^53–58^.

In addition to the selection of a driver of acute activation, an objective real time physiologic readout of an attack, such as hypotension, is also needed for a model of acute HAE attacks. Telemetry devices can be implanted in mice to allow for real time measurement of blood pressure^59^.

In this article, we present the first in vivo murine model to mimic acute HAE attacks. Attacks were induced by IV injection of a silica nanoparticle (SiNP) suspension^60–64^. Blood pressure was measured in real time, in conscious and untethered mice using implanted telemetry. SiNP injection induced a rapid, reversible decrease in blood pressure, in the presence of angiotensin converting enzyme (ACE) inhibition. The robust and reproducible murine model reported here will facilitate understanding and development of interventions to prevent and treat acute HAE attacks.

## Methods

### Animals

Male and female serping1−/− mice as well as wild-type C57BL6J mice (Age 4–6 months) were used. The serping1-deficient mouse was generated by Deltagen Inc. (San Mateo, CA), applying homologous recombination using ES cells. A Lac-Z/neo target vector was introduced to disrupt the serping1 (Gene#763, Serpin 1, Genbank Accession #Y10386 gi:1772997), using mouse ES cells derived from 129/Olahsd mouse sub strain (line #5961). Southern Blot was used to confirm correct targeting. serping1−/− and serping1+/+ genotypes were verified by PCR amplification by genomic DNA using primers spanning the LacZ/Neo insertion site (Supplemental Fig. S1) The resulting serping1−/+ males and females were backcrossed to C57BL6J to generate congenic strains (Charles River Laboratories). In the FDA vivarium, the serping1−/+ animals were interbred for more than 20 generations to obtain serping1−/− animals.

We backcrossed the Serping1−/− (129/Olahsd /C57Bl6J background) congenic mouse strain to wild type C57BL6/J (Stock#000664; Jax Laboratories) for 12 generations to generate the serping1−/− animals on a pure C57Bl6 background. The genotype and pedigree of the backcrossed mice were tested using the GigaMUGA platform (University of North Carolina at Chapel Hill). A western blot was used to verify the knockout animals lack expression of an intact C1 inhibitor protein (Supplemental Fig. S2). All experimental animals were maintained in accordance with the Institutional Animal Care and Use Committee (IACUC) and White Oak Consolidated Animal Program. The study was carried out in compliance with the ARRIVE guidelines.

### Surgical implantation using wireless telemetry

Implantable transmitter HD-X11 (Data Sciences International, St. Paul, MN) is designed to simultaneously measure mean arterial pressure (MAP), temperature, locomotor activity and ECG at 10 s intervals allowing for accurate time-resolution of treatment-related effects. Animals were anesthetized with Isoflurane (by vaporizer—3–5% for induction, 1–3% for maintenance, nose cone). Buprenorphine SR (sustained release) Lab at 1 mg/kg was used subcutaneously on the day of the surgery. Because of its sustained release formulation, it can provide analgesic effect for ~ 48–72 h. post-surgery, thereby reducing handling of post-operative animals. The HD-X11 transmitters were implanted in the intraperitoneal cavity, with the fluid filled catheter inserted into the left carotid artery, according to manufacturer’s instructions. After securing the ECG leads on the body muscle, the skin was sutured using non-absorbable 5-O sutures. Post-operative animals were administered warmed Lactate Ringer solution (LRS). Mice were placed in individual cages, on heating pads, until the animals regained independent mobility. All procedures conducted on mice were approved by the IACUC of Division of Veterinary Services (DVS) at the Food and Drug Administration, Silver Spring, Maryland. Raw data collection and analysis were implemented using Dataquest ART (v. 4.3). A 7-day period of post-surgery data collection was performed to record baseline data. Later during experimental dosing, BP data were collected at 10 s intervals for 1 h preceding the intravenous injection and compared to MAP data collected during a 1-h post injection period (Supplemental data S3–S5).

### Efficacy studies in mice

After 1 week of post-surgical recovery serping1−/− mice (3–4 animals/group) received 100 µl of 0.25 mg of SiNPs (Silica Nanospheres, mean diameter 50 nm, nanoComposix, San Diego, CA) or saline via bolus intravenous tail injections. The animals were pre-treated with angiotensin converting enzyme inhibitor (ACEi), Captopril (12.5 mg; Mylan, WV) in their drinking water (0.01 mg/ml), 24 h prior to injection^65^. Based on established values for mean daily water consumption^66^ each animal received an approximate captopril dose of 2.5 mg/kg/day. Individual animals were subjected to up to three separate interventions under different experimental conditions (e.g. SiNP, Captopril, saline, SiNP+ antagonist). Each intervention was separated by a 1-week recovery period.

A control group of C57BL6J females (Jax# 00064) was used to compare the effect of SiNPs and ecallantide (10 mg/ml, Dyax, Burlington,MA) exposing the wild-type (WT) females to same conditions as their serping1−/− female counterparts.

### Effect size calculations

MAP was continuously monitored (every 10 s) via Dataquest ARTv4.3 for each animal from 30 min prior to experimental intervention (injection of SiNPs) until 60 min after injection. The first post-injection blood pressure reading was defined as t = 0. Baseline MAP was defined as the mean blood pressure from t = − 15 to t = − 10 min. For each animal, all post-intervention MAP values were expressed relative to the mean baseline MAP value. The relative MAP at time point n (RMAP_n_) was calculated as: MAP_n_/MAP_baseline_.

The effect of SiNP on MAP was calculated at each time point by subtracting the MAP at t = 0 from the measured MAP. The net RMAP at time point n (Net RMAP_n_) was expressed as: Net RMAP_n_ = RMAP_n_ − RMAP_t=0_. The total effect of SiNP on blood pressure was calculated as the sum of Net RMAP values from t = 0 to 12 min. MAP values generally returned to baseline values by t = 12 min. Only those time points in which RMAP_n_ was ≥ 10% below RMAP_t=0_ were included in this calculation in order to capture physiologically relevant effects.

### Statistical analysis

Statistical analyses were conducted using GraphPad PRISM (8.0 GraphPad Software, San Diego, CA). All data were expressed as the Mean ± SD. Assessment of the differences between groups of treatments were performed using 2-way ANOVA (Analysis of Variance) and Bonferroni’s post-hoc tests comparing all group means. A *P*-value of < 0.05 was considered statistically significant. At least nine animals were evaluated for each experimental group; However, fewer animals were evaluated for some controls. Animal numbers are included in figure legends.

## Results

### SiNP injection models acute HAE attack in serping1−/− mice

**Figure 1:**
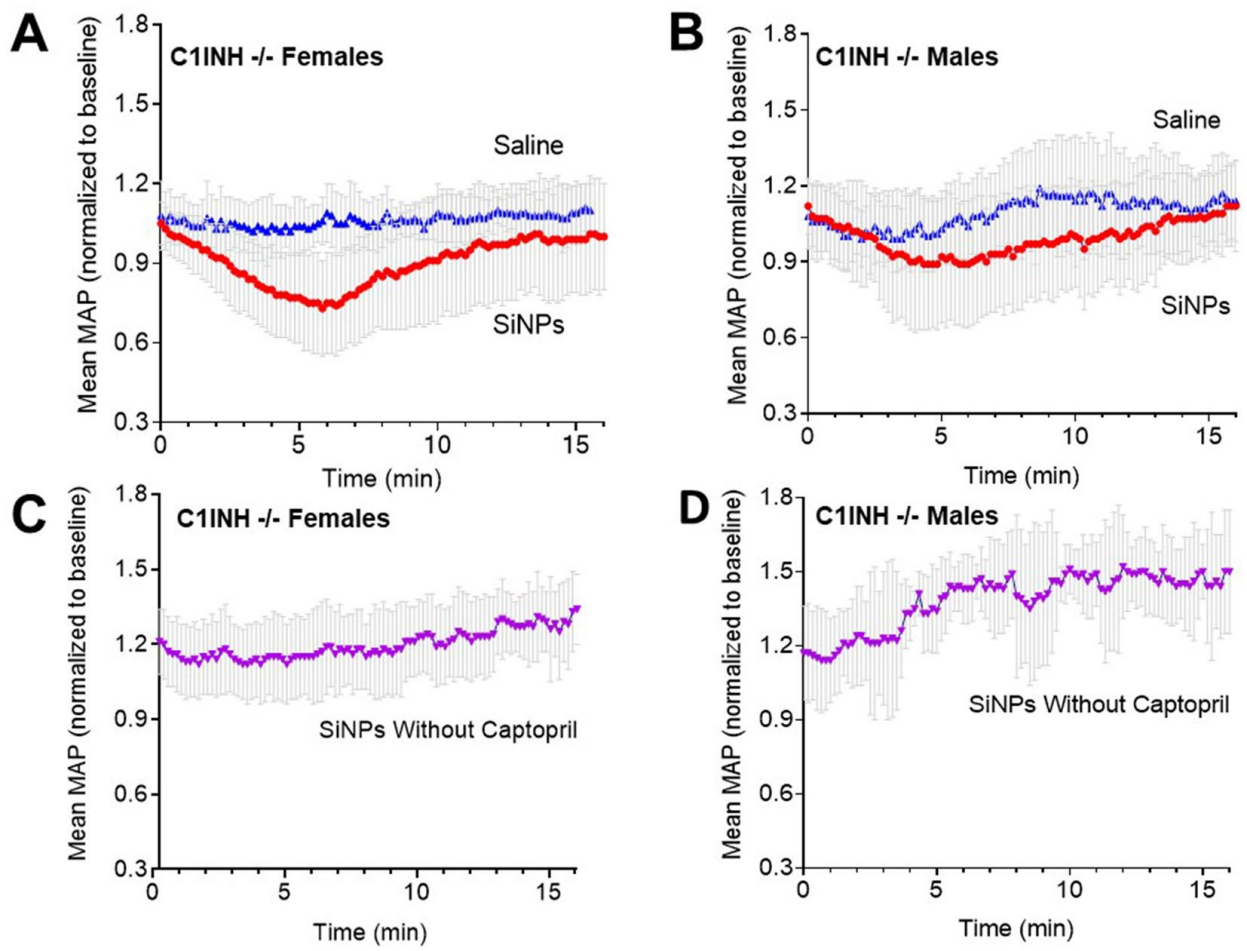
In Vivo stimulation of the contact pathway with SiNPs in serping1−/− mice evokes acute HAE-like attacks in the presence of Captopril. Bolus IV injection of SiNPs (0.25 mg/100 µl) at t = 0 in captopril treated serping1−/− females; n = 18 (**A**) and males; n = 12 (**B**) evoked a rapid and reversible decrease in MAP. When serping1−/− females (n = 9) and males (n = 6) were injected with a single bolus IV injection of saline (vehicle), in the presence of captopril, no change in MAP was reported (**A**, **B**). Bolus IV injection of SiNPs (0.25 mg/100 µl) in the absence of captopril had no such effect on MAP in serping1−/− females; n = 6 (**C**) or males; n = 3 (**D**). Data points represent mean ± SD of normalized MAP.

We chose silica nanoparticles (SiNPs) to simulate acute HAE attacks in our in vivo murine model as silica nanoparticles (SiNPs)^52^ have been shown to activate FXII^53–58^. A dose of 0.25 mg/100 µl of SiNPs was selected based upon published toxicity data in mice^67,68^. With ACEi captopril pretreatment^69^, intravenous SiNP injection induced a reversible decrease in blood pressure in serping1−/− females and males (Fig. 1A,B). When the same set of mice with captopril pretreatment were intravenously injected with saline, they failed to demonstrate any drop-in MAP (Fig. 1A,B). SiNP injections, in the absence of captopril pretreatment, did not lead to blood pressure drops in serping1−/− males or females (Fig. 1C,D); in fact, a blood pressure increase post-injection was observed. Examples of individual animal data are in the “Supplementary Materials S1” (Supplementary Figs. S3 and S4). In addition to decreased blood pressure, observation of captopril and SiNP treated animals revealed markedly decreased locomotor activity relative to captopril and saline treated controls (Supplementary Fig. S5). These observations reversed on the same time course as the measured blood pressure changes.

### The plasma kallikrein inhibitor ecallantide inhibits SiNP-induced blood pressure decrease

**Figure 2:**
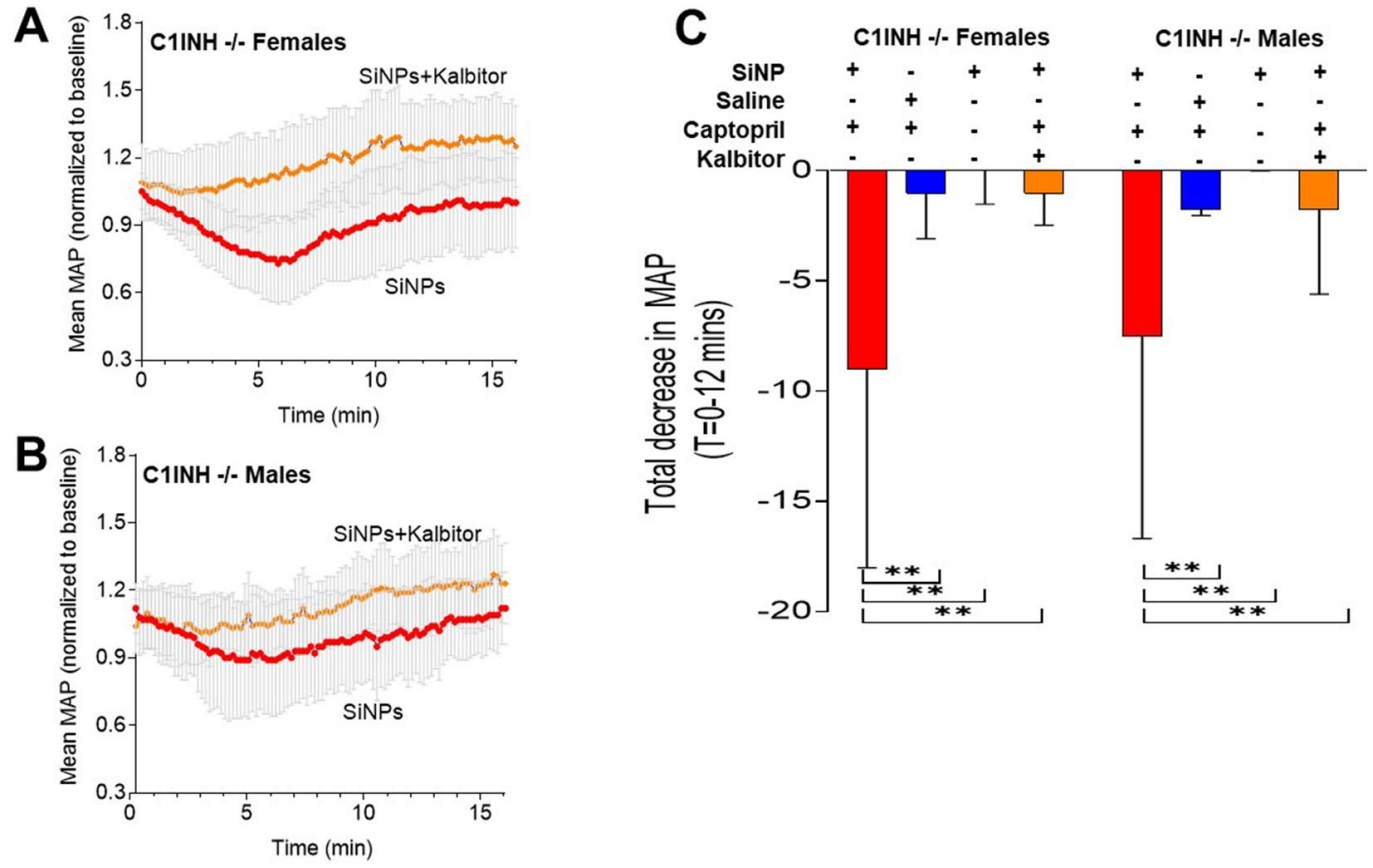
Ecallantide (Kalbitor) ameliorates the SiNP-induced MAP decrease in captopril-treated Serping1−/− mice. (**A**, **B**) Kalbitor (100 µg), injected immediately prior to a bolus IV injection of SiNPs (0.25 mg/100 µl) at t = 0, inhibits the SiNP-induced decrease in mean MAP in both (**A**) serping1−/− females (n = 9) and (**B**) serping1−/− males (n = 9). (**C**) The histogram compares the total decrease in MAP (t = 0—12 min) in serping1−/− female and serping1−/− male mice as per the Materials and Methods, with four different interventions. Data are expressed as mean ± SD. Statistical analysis was performed using 2-way ANOVA with Bonferroni’s post-hoc tests SiNPs in the presence of captopril vs. saline in the presence of captopril *P = 0.002; SiNPs in the presence of captopril vs. SiNPs in the absence of Captopril **P = 0.007; SiNPs in the presence of Captopril vs SiNPs + Ecallantide in the presence of captopril **P = 0.002.

We used ecallantide (Kalbitor), an FDA-approved HAE therapy, to evaluate the model’s ability to respond to HAE therapeutics. Ecallantide (100 µg/100 μl) was injected immediately prior to SiNP injection in captopril-treated mice. Ecallantide blocked the SiNP induced MAP decrease in both captopril pretreated male and female serping1−/− mice (Fig. 2A,B) Silica vs Saline P = 0.002; Silica + Captopril vs Silica Without Captopril P = 0.007, and Silica vs Kalbitor, P = 0.002. The results of all the interventions were integrated over time as per the “Materials and methods” in Fig. 2C. Even though the results indicated a potential trend towards sex-based difference in response to SiNP, post-hoc testing did not indicate significant differences between the sexes (P = 0.87).

### Wild-type females failed to show a significant effect of ecallantide treatment on captopril and SiNP-induced in blood pressure drops

**Figure 3:**
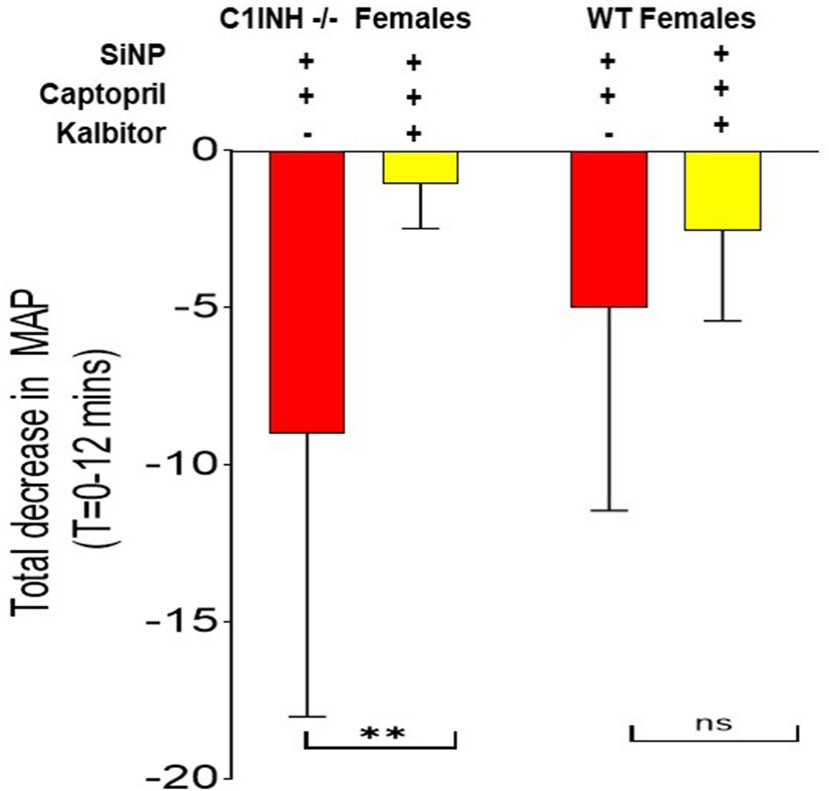
The Serping1−/− murine model is more sensitive than C57BL6 WT mice for assessing treatment of acute HAE-like attacks. Comparison of the integrated effect size, as per the Materials and Methods, of ecallantide treatment of SiNP-induced blood pressure drops in Serping1−/− (n = 18) and WTC57Bl6/J female mice (n = 9). There is significant amelioration of SiNP-induced MAP decrease with ecallantide treatment in Serping1−/− females (P = 0.01) as compared to the C57Bl6 females (NS, P = 0.86). Assessment of the differences between groups of treatments were performed using 2-way Analysis of Variance (ANOVA) with Bonferroni’s post-hoc test (used to determine the significance of mean MAP change of all groups against each other) with *P < 0.02; NS = non-significant. Data are expressed as mean ± SD.

Ecallantide inhibited the SiNP-induced MAP decrease in serping1−/− females (P = 0.01) but not in C57Bl6J females (P = 0.86) as shown in Fig. 3. Assessment of the differences between groups of treatments was performed using two-way Analysis of Variance (ANOVA) with Bonferroni’s post-hoc test. SiNP-induced blood pressure decreases were nominally smaller in WT C57Bl6J mice relative to serping1−/− animals. These data support the conclusion that serping1−/− mice pretreated with captopril and injected with SiNP are a more sensitive model for HAE-like attacks and treatments compared with WT mice under the same conditions.

## Discussion

Hereditary angioedema is a rare genetic disease with significant mortality that is generally caused by mutations in the serping1 gene leading to either reduced levels (Type I HAE) or abnormal function (Type II HAE) of the C1-esterase inhibitor. Although there are murine models for HAE, there has not been an HAE animal model for acute attacks of angioedema. In this study, we describe a reliable and reproducible in vivo murine model that mimics acute HAE attacks. In this model, HAE-like attacks are successfully mitigated by the FDA-approved HAE therapeutic, ecallantide.

The role of bradykinin generation in HAE murine models has been demonstrated in earlier studies^32^. The importance of captopril, an ACE inhibitor, in this model enhances the role of bradykinin as ACE catabolizes bradykinin rapidly and ACE inhibitors can increase the levels and duration of kallikrein generated bradykinin^70–72^.

**Figure 4:**
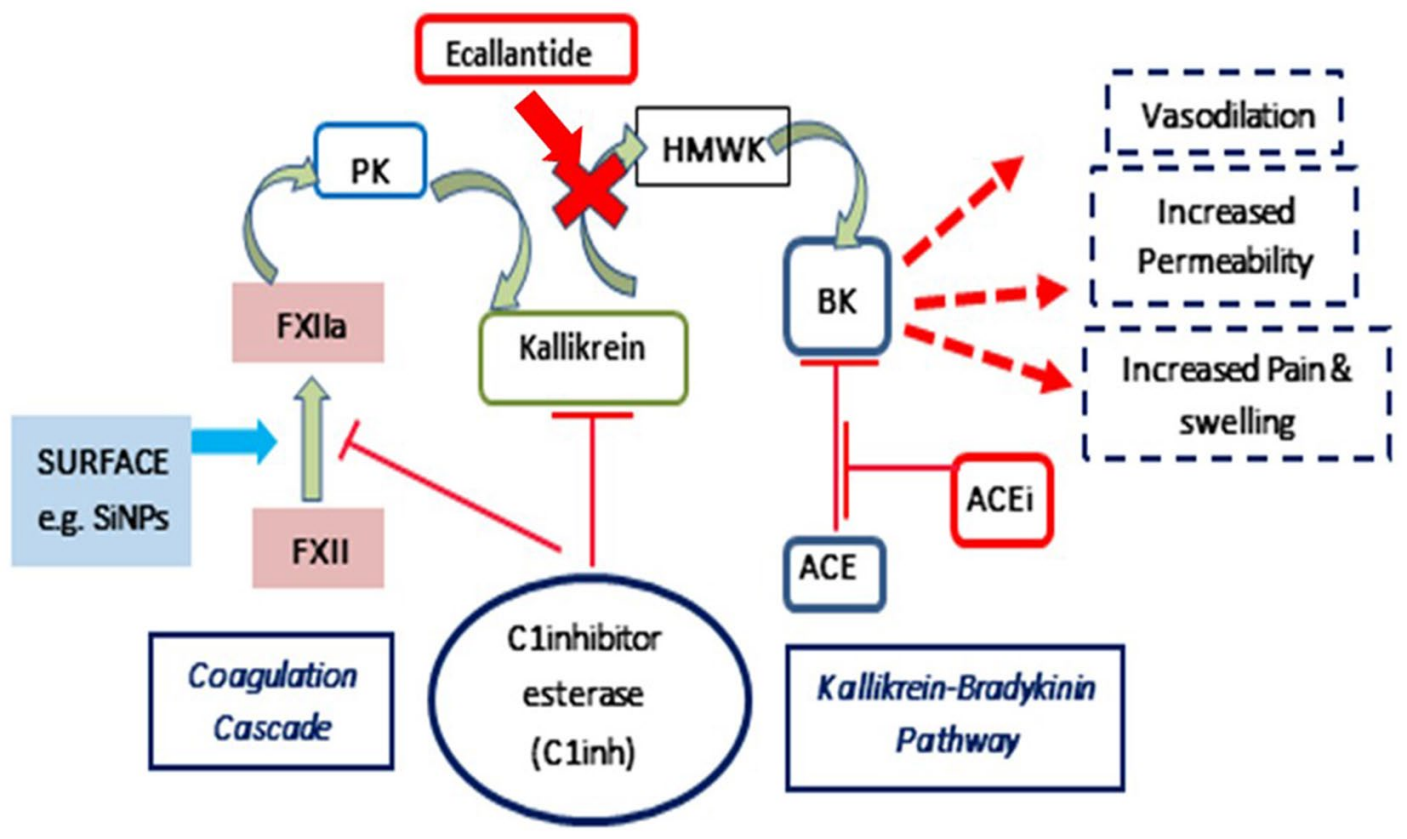
Contact system pathways with the kallikrein-bradykinin pathway and Serping1. Serping1 acts as the primary plasma inhibitor of the contact-kinin system cascades. In the absence of Serping1, FXIIa converts prekallikrein (PK) to kallikrein, which in turn cleaves high molecular weight kininogen (HK) and releases bradykinin (BK). Bradykinin is degraded by ACE and other plasma enzymes. The levels and duration of bradykinin in plasma impacts vascular permeability and vasodilation, leading to decreases in blood pressure and swelling; a hallmark for HAE. Ecallantide acts as a plasma kallikrein inhibitor and limits the production of bradykinin from HK.

In humans, HAE attacks occur in the absence of ACE inhibitors^69^. The need for ACE inhibition in this model may reflect differences in bradykinin regulation between mice and humans. Human HAE occurs as an autosomal dominant genetic inheritance pattern and affected patients have residual levels of functional serping1. There does not seem to be a known syndrome with complete absence of serping1 in humans. Mice with complete loss of serping1 are viable and do not seem to have spontaneous attacks^32^. These mice may have additional regulation of kallikrein-mediated bradykinin generation and/or bradykinin degradation (Fig. 4), for example, non-ACE pathways of bradykinin clearance. There may also be other compensatory mechanisms. Of note, in the absence of ACE inhibition, the post-SiNP injection trend of increased blood pressure may reflect other regulatory systems in mice. Such differences may explain the need for ACE inhibition in this model.

A murine model of C1 inhibitor deficiency with a heterozygous genotype and increased vascular permeability despite has been developed^73^. This model used CRISPR‐Cas9 to generate a truncated protein rather than inserting another construct into the Serping1 gene as we and others^32^ have done. Our model targets exon 4 and allows for antigen expression but deletes an ~ 75 KDa C1 inhibitor reactive band compatible with glycosylated C1 inhibitor (Supplemental Fig. S2). We cannot rule out alternative splice variants and there is some MAP impact of our approach in WT mice; However, we do observe a difference between the KO mice and WT mice (Fig. 3).

The heterozygote model better mimics heterozygote human disease; However, the model did not exhibit spontaneous attacks. A rat model of HAE did have some spontaneous intestinal swelling; however, this model was based on bradykinin overexpression rather than Serping1 mutations. Directly mimicking all features of human disease is challenging as human disease phenotypes may be dependent on specific mutations^74^. as well as species-specific characteristics of the contact system. Our approach of using real-time MAP monitoring and an inducer of acute attacks, such as SiNP, could be applied across a range of HAE animal models and increase the utility of such models.

This model can also further explore sex-related effects in HAE with additional studies in larger numbers of mice, in both serping1−/− and WT animals. The evaluation of sex differences is important because female sex hormones, mainly estrogen, may impact bradykinin generation^75–77^. This robust in vivo model may also facilitate further study of the mechanisms that can induce acute HAE attacks. In summary, the serping1−/− murine model is the first model to evoke acute HAE-like attacks and can also be used to study approved HAE drugs (i.e. danacrine, icatibant, cinryze, lanadelumab)^78–82^, and support development of future targeted novel drug therapies for HAE prophylaxis.

## Supplementary Information

Supplementary Information.
