# Supplementary material for: A novel murine in vivo model for acute hereditary angioedema attacks
Source: Sci Rep. 2021 Aug 5;11:15924. doi: 10.1038/s41598-021-95125-0 (PMC8342443; doi:10.1038/s41598-021-95125-0)
Supplement: Supplementary file 1 — Supplementary Information. [file 41598_2021_95125_MOESM1_ESM.docx]

SUPPLEMENTAL DATA

A NOVEL MURINE *IN VIV*O MODEL FOR ACUTE HEREDITARY ANGIOEDEMA ATTACKS

**Sujata Bupp^1^, Matthew Whittaker^2^, Mari Lehtimaki^1^, JuMe Park, Jessica Dement- Brown, Zhaohua Zhou and Steven Kozlowski^1^§**

**Affiliations**

1. Office of Biotechnology Products, Office of Pharmaceutical Quality, Center for Drug Evaluation and Research, US Food and Drug Administration, Silver Spring, Maryland 20993
2. Office of New Drugs, Center for Drug Evaluation and Research, US Food and Drug Administration, Silver Spring, Maryland 20993

**Supplemental Figure S1 C1inh Knockout Strategy and Detection**

**A**

| \| **LacZ-Neo** \| \| --- \| \| 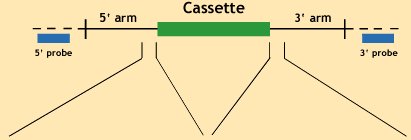 \| |
| --- | --- | --- |
|  |
|  |
| \| **5'>**[AAAAAAATAAATTAAATTAAA TAAATAAATAAGGAAAAAGTGAAA CCAGAACCTATGGGAAGTGTGCTC TCTCTCATACTTGCCCTTTGAAGT AGGGGAGGACTGCTCAGGAGACTC ACACTTTCTTGCCTTGTCCTGCAT CTCAGATCTGGCCATAAGGGACAC CTATGTGAATGCATCTCAGAGCCT GTATGGAAGCA](JavaScript:PopWindow(document.forms[0].hdnSeqType.value,%20document.forms[0].hdn5GenomicArmSeq.value))**<3'** \| **5'>**[ACCAACCATAAGATCCGCAAG CTGCTGGGCAGCCTGCCTTCTGAC ACCTGCCTCGTCCTTCTCAATGCT GTCTACTTGAGTGGTAAGGGAAAC ACCAGGCAGGTAGTGTGCCCTGCC CAATGCTGTTCTGCCTCCCCTTCT GTCTCCAGGCTCAGAGTTCCACAG ACACATTCCCATGTGACTGACAGT CCTACCCTTCC](JavaScript:PopWindow(document.forms[0].hdnSeqType.value,%20document.forms[0].hdn3GenomicArmSeq.value))**<3'** \| \| --- \| --- \| |

**B C**


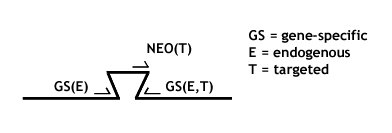

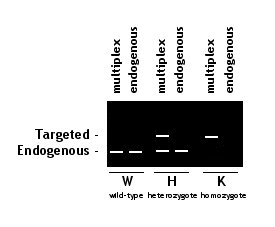


**D**

| **Type** | **ID** | **Sequence** |
| --- | --- | --- |
| GS(E,T) | 53879 | ACTACCTGCCTGGTGTTTCCCTTAC |
| NEO(T) | 3196 | GGGTGGGATTAGATAAATGCCTGCTCT |
| GS(E1) | 53878 | TTTGAAGTAGGGGAGGACTGCTCAG |

**E**


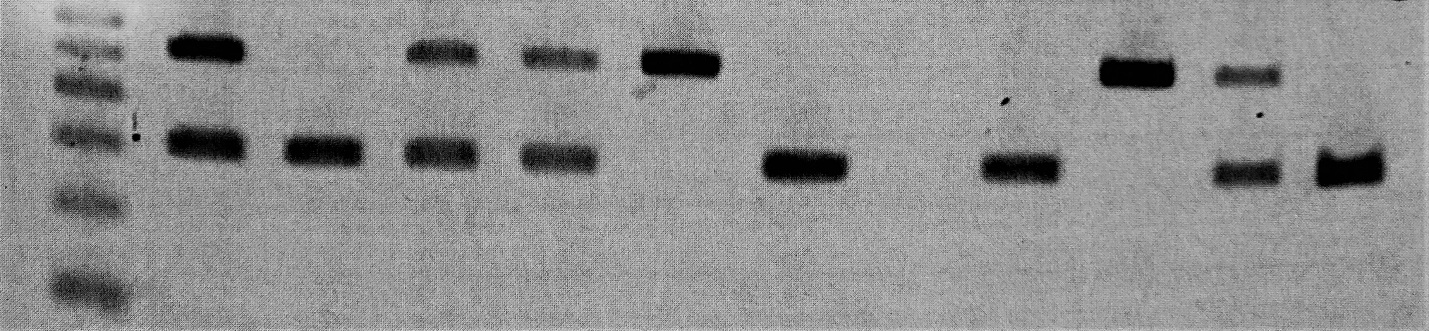


**W K H W**

Targeted

Endogenous

**Supplemental Figure S1 Legend:** The Deltagen strategy for generation of the mouse model is shown in S1A. The LacZ-Neo cassette was inserted in the 4^th^ exon of the C1 inhibitor gene with the flanking sequences as shown in S1A. Genomic DNA from the recombinant ES line was assayed for homologous recombination using polymerase chain reactions (PCRs). Amplified DNA fragments were visualized by ethidium bromide staining following agarose gel electrophoresis. The test PCRs employed the gene-specific (GS) primer, which lies outside of and adjacent to the targeting vector arm, paired in succession with one of three primers in the insertion fragment (S1A). The "DNA sample control" employed a primer pair intended to amplify a fragment from a non-targeted genomic locus. The "positive control" employed the GS primer paired with a primer at the other end of the arm. The table below the gel image (S1D), lists the primers (numbered) used in each PCR in base pairs (bp) The diagrams depict the anticipated gel image (S1C) as well as the relative positions of the PCR primers (S1B). An example PCR used in screening animals is shown in S1E (W- Wild Type, H-Heterozygote, K-Knock out for C1inhibitors).

**Supplemental Figure S2. Detection of C1Inh by Western**


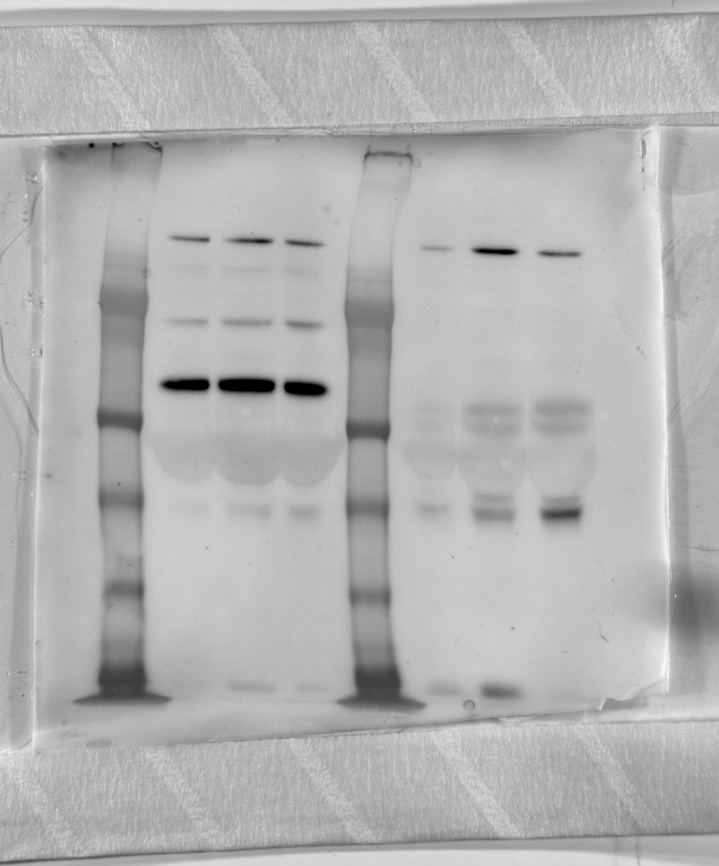
 **A M +/+ +/+ +/+ M -/- -/- -/-**

**98 kDa**

**62 kDa**

**B M +/+ +/+ +/+ M -/- -/- -/-**


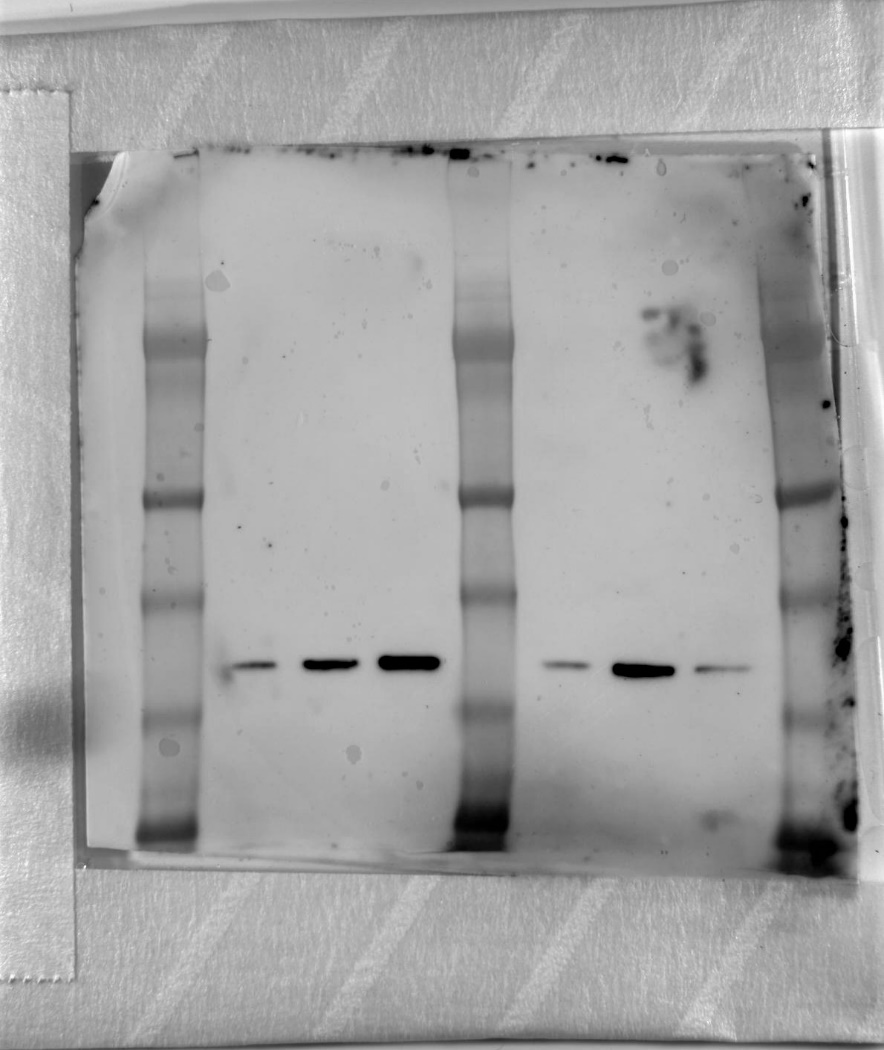


**49 kDa**

**38 kDa**

**Supplemental Figure S2 Legend. Blot Analysis in Wild Type and Knockout Mice**

The LacZ-Neo insertion in exon 4 allows for expression of C1 inhibitor antigen, especially at the N terminus. Thus, we evaluated the knockout mice for C1 inhibitor using a western blot method. Sera from 3 wild type C57Bl6J (+/+) and three C1inh knockout mice (-/-) were evaluated for the presence of C1inhibitor protein using a western blot method. A band of approximately 75,000 Daltons was detected in the wild type mice that was not observed in the knockout mice using a monoclonal anti-Serping1 (S2A). The molecular weight markers are indicated (M). Actin at 43,000 Daltons is detected in the same samples from both wild type and knockout mice (S2B). The photographs were not manipulated and not cropped beyond the edge of the blots.

**Western Blot Analysis Method**

Mouse serum from female C1inh KO and C57Bl6J (1:100 dilution) was added to Loading buffer and reducing agent (LDS Sample Buffer; Sample Reducing Agent; NuPAGE, ThermoFisher) and boiled for 5 minutes at 95 ⁰C. Samples were separated on an 8% Bis-Tris gel (Bolt Bis-Tris, ThermoFisher), at 165V for 45 minutes, in MOPS buffer. Proteins were then transferred to nitrocellulose membrane using iBlot gel transfer device (iBlot Invitrogen, Life Technologies). Membranes were blocked for 1hr at RT in 5% milk dissolved in TBS-0.01% Tween 20 (TBST).

After blocking, membranes were incubated with mouse monoclonal antibody C1INH B-11 (sc-377062; Santa Cruz Biotechnology, Inc), at 4⁰C overnight. Blots were then washed with TBST-0.01%Tween20 (TBST) and C1 inhibitor was detected with C1Inh B-11(sc-377062 HRP; Santa Cruz, Biotechnology, Inc) conjugated to horseradish peroxidase. Blots were extensively washed with TBST and developed with chemiluminescent HRP substrate (Immobilon Western Chemiluminescent HRP substrate; WBKLS0500, Millipore, Sigma, USA). The blots were then analyzed using an ImageQuant 800 GxP (Cytivia 2021, Amersham^,^ USA). Western blots performed with labeled anti-C1inh in the absence of an unlabeled anti-C1inh incubation had a ~75 kDa band in wild type mice that was absent in knockout mice; However, there was a much higher background signal.

Beta actin was evaluated as a control. The beta Actin Loading Control Monoclonal Antibody (BA3R) (MA5-15739, Abcam, USA) was used following the above Western Blot analysis protocol. Detection was with the HRP conjugated beta Actin Monoclonal Antibody (BA3R), HRP (MA5-15739-HRP, Abcam).

**38 kDa**

**Supplemental Figure 3 Mean Arterial Pressure in Individual Saline Treated Mice**


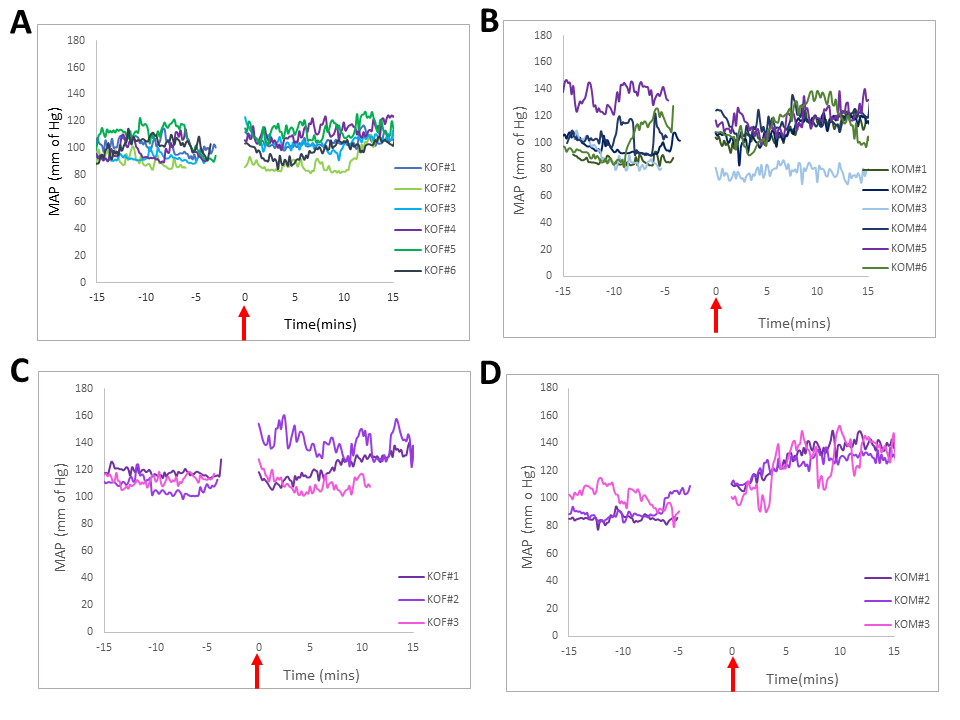
**Supplemental Figure 3 Legend**. Real-time data Analysis from DataQuest ARTv4.3 showing the effect of saline (control) and SiNPs injected in in both Serping1-/- Females and Males. In agreement with the data of Figure 1, C1inhKO females (S1 A) and males (S1 B) injected with saline, in the presence of captopril, fail to show a drop-in MAP. In the absence of captopril, when SiNPs were administered via tail vein injection, no significant drop in MAP was observed for females (S1 C) or males (S1 D). The first MAP reading after each injection was defined as T = 0. Blood pressure measurement was calculated at each time points subtracted from T=0, prior to and post-intervention. The red arrow denotes the time (t=0) when the animals receive their single dose of interventions

**Supplemental Figure S4 Mean Arterial Pressure in Individual SiNP Treated Mice**


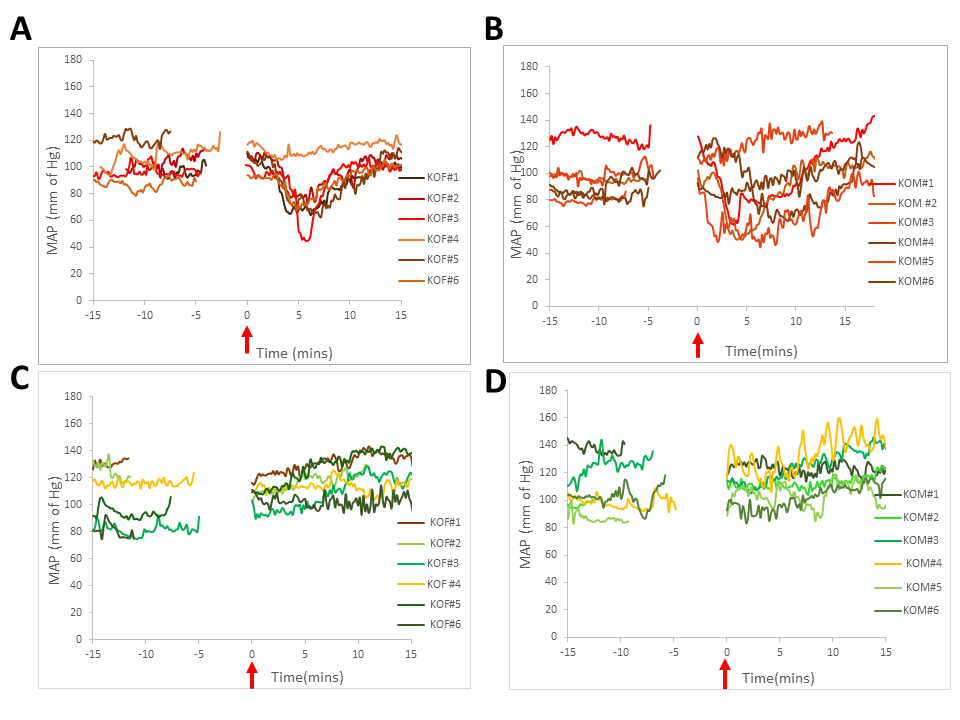
**Supplemental Figure S4 Legend**. Real-time Data Analysis from DataQuest ARTv4.3 showing the Effect of SiNPs and Ecallantide (Kalbitor) in the presence of Captopril in Serping1-/- Females and Males. When C1inhKO females (S2 A) and males (S2 B) are injected with SiNPs, a substantial drop in MAP was observed. Plasma kallikrein inhibitor, ecallantide (Kalbitor) markedly impeded blood pressure drops, for both females (S2 C) and (S2 D) males. The red arrow denotes the time (t=0) when the animals receive their single bolus of intervention.

**Supplemental Figure S5 Activity Analysis**


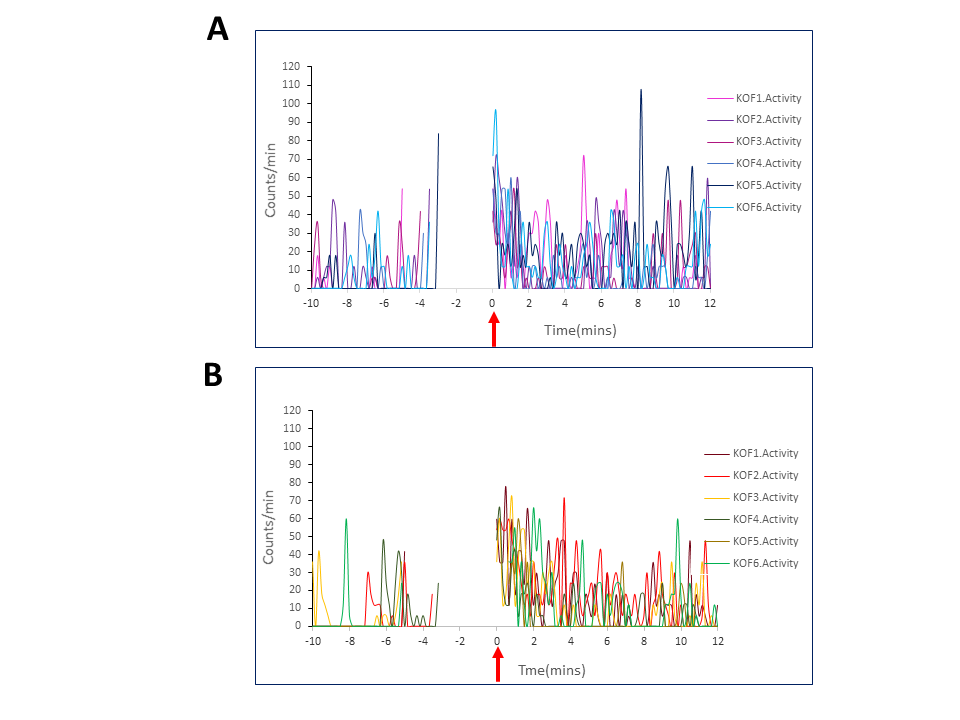
**Supplemental Figure S5 Legend** Real-time data analysis from DataQuest ARTv4.3 showing the effect of saline and SiNPs in the presence of Captopril in Serping1-/- Females. When the same C1InhKO females, pretreated with captopril were, are injected with saline (S3 A), they were noticeably more active than when injected with SiNPs (S3 B). The red arrow denotes the time (t=0) when the animals receive their single dose of interventions

**Supplemental Data**

**Supplemental Table S1** **Calculation of Murine Ecallantide Dose based on Body Surface Area.**

| **Approved human Dose (mg)** | **Approved dose (mg/kg) ^1^** | **Conversion factor for murine dose** | **Murine dose (mg/kg)** |
| --- | --- | --- | --- |
| **30** | **0.5** | **12.3** | **6.2** |

^1^ Based on 60 kg human weight

The approved clinical dose of ecallantide (Kalbitor) is 30mg. The murine equivalent dose is calculated by multiplying the human dose by 12.3. Thus, for a mouse weighing 0.02 kg, the murine ecallantide dose is approximately 120 µg.

This is based on: Guidance for Industry- Estimating the Maximus Safe Starting Dose in Initial Clinical Trials for Therapeutics in Adult Healthy Volunteers. Food and Drug Administration, U.S. Department of Health and Human Services. July 2005.
